# Supplementary material for: Concern noted: a descriptive study of editorial expressions of concern in PubMed and PubMed Central
Source: Res Integr Peer Rev. 2017 May 27;2:10. doi: 10.1186/s41073-017-0030-2 (PMC5526611; doi:10.1186/s41073-017-0030-2)
Supplement: Supplementary file 1 — Search strategies. (DOCX 24 kb) [file 41073_2017_30_MOESM1_ESM.docx]

**Additional file 1. Search strategies.**

Concern noted: a descriptive study of editorial expressions of concern in PubMed and PubMed Central. A PubMed Commons post-publication project: https://osf.io/8xbqy/

**1. PubMed and PMC (PubMed Central)**

PubMed and PMC were searched from inception with this string:

(“expression of concern”[all fields] OR “notice of concern”[all fields] OR “note of concern”[all fields] or "statement of concern"[all fields]) OR ((expression*[ti] OR statement*[ti] OR note*[ti] OR notice*[ti]) AND concern*[ti])

The following additional searches were run in PMC:

- "expression of concern"[filter]
- "has expression of concern"[filter]
- correction[Filter] AND (editor OR editors) AND (express) AND (concern OR concerns) AND (wish OR issue)

Date of last update search in PubMed and PMC: 22 August 2016.

**2. Retracted expressions of concern (EEoCs)**

After the master list of EEoCs was finalized, PMC was searched using the “is retracted” filter and PubMed was searched by one author (DJ) for withdrawals or retractions of EEoCs on 7 December 2016 using these searches:

- <EEoC PMIDs> AND (remov* or withdraw* or retract* or hasretractionin or retracted publication[pt])
- <EEoC PMIDs> AND hascommentin

**3. Google Scholar**

Google Scholar (GS) was searched to identify EEoCs for publications in PubMed and PMC which were not submitted to PubMed/PMC. (PubMed records are generated for publications submitted to PMC for non-MEDLINE journals.)

Searches were undertaken by one author with “patents and citations” de-selected. “Eurosurveillance” entries were ignored, as frequent results were returned where “expression of concern” was a news item on the website, unassociated with the individual publication.

GS does not enable download of search results, but the number of results and the number screened were recorded. Data saturation was determined to be either five consecutive page results with no EEoC, or the first 1,000 search results. Searches were:

- “editorial concern”
- “expression of concern”
- “expression of concerns”
- “note of concern”
- “notice of concern”
- “statement of concern”

Searches were undertaken by HB and MV on 18 August 2016.

**4. Retraction Watch**

The archive of posts tagged “Expression of concern” at the blog, Retraction Watch [1] was searched by one authors (DJ) on 1 November 2016, with confirmation by another (MV):

<http://retractionwatch.com/category/by-reason-for-retraction/expression-of-concern/>

**5. Publisher websites**

Websites for the top 5 publishers in social sciences and humanities and in natural and medical sciences in 2013 were selected; 4 publishers appear in the top 5 for both subject areas, resulting in 6 publishers for review [2]. Publishers with web interfaces that supported search of multiple journals and had at least 1 journal with an EEoC that had been identified but not found by PubMed/PMC searches were also selected. One author (MV) searched these 9 publisher websites using the search term “expression of concern” in September and November 2016:

- American Chemical Society
- Bentham Science
- Cell Press
- Oxford Journals
- ScienceDirect
- SpringerLink (also searched “notice of concern”)
- Sage Publications
- Taylor & Francis (also searched “notice of concern”)
- Wiley Online Library

**6. Reference searching**

Previous studies and listings of EEoCs known to the authors [3-7] were independently hand-searched by pairs of authors (HB, DJ, MV) in August and December 2016.

**7. Journal websites**

For all journals where an EEoC was identified either through searches (16 journals) or reading associated with the project (1 journal) rather than PubMed/PMC searches, the journal website was searched with the terms used in the EEoC(s) which had been identified. In 2 cases, searching the target journal website also searches other journals of a specialty society (Journal of Physiology and Neurology). In addition, some journals were also searched using Google or PMC. Searches were undertaken by either HB or MV, between August and December 2016. This resulted in searches of these 17 target journals:

- Blood
- CNS and Neurological Disorders - Drug Targets
- Drug Delivery and Translational Research
- Experimental Physiology
- Expert Opinion on Therapeutic Targets
- Expert Opinion on Biological Therapy
- Immunology
- Journal of Biological Chemistry
- Journal of Clinical Investigation
- Journal of Physiology
- Journal of the American College of Cardiology
- Neurology
- Nucleic Acids Research
- PeerJ
- Pharmacological Reviews
- Stem Cells
- The Oncologist

**References**

1. Retraction Watch. http://retractionwatch.com/. Accessed 01 Nov 2016.
2. Larivière V, Haustein S, Mongeon P. The Oligopoly of Academic Publishers in the Digital Era. PLOS ONE. 2015;10:e0127502.
3. Noonan BM, Parrish D. Expressions of concern and their uses. Learned Publishing. 2008;21:209-213.
4. Grieneisen ML, Zhang M. A comprehensive survey of retracted articles from the scholarly literature. PLoS One. 2012;7:e44118.
5. Marusic A, Katavic V, Marusic M. Role of editors and journals in detecting and preventing scientific misconduct: strengths, weaknesses, opportunities, and threats. Med Law. 2007;26:545-566.
6. Roig M. An exploratory content analysis of Expressions of Concern. In: Proceedings of the 4th World Conference on Research Integrity. Research Integrity and Peer Review; 2016;1 Suppl 1:CS04.3.
7. Scott-Lichter D, Editorial Policy Committee, Council of Science Editors (CSE). CSE’s White Paper on Promoting Integrity in Scientific Journal Publications. 2012. <http://cseditors.wpengine.com/wp-content/uploads/entire_whitepaper.pdf>. Accessed 16 Aug 2016.
