# Supplementary material for: Concern noted: a descriptive study of editorial expressions of concern in PubMed and PubMed Central
Source: Res Integr Peer Rev. 2017 May 27;2:10. doi: 10.1186/s41073-017-0030-2 (PMC5526611; doi:10.1186/s41073-017-0030-2)
Supplement: Supplementary file 7 — Definitions and key for content analysis of EEoCs and post-EEoC events. (DOCX 111 kb) [file 41073_2017_30_MOESM7_ESM.docx]

**Additional file 7. Definitions and key for content analysis of editorial expressions of concern (EEoCs) and post-EEoC events.**

Concern noted: a descriptive study of editorial expressions of concern in PubMed and PubMed Central. A PubMed Commons post-publication project: https://osf.io/8xbqy/

**Key:**

R = Reason for expressing editorial concern or for post-EEoC event.

C = Current status of editorial expression of concern.

Im = Images are the, or a, reason for the EEoC or post-EEoC event.

Adj = Adjudication in the case of a finding of research misconduct (R 1 – 4).

Inv = Official investigation by employer or funding agency.

| **Code** | **Descriptor** | **Definition** |
| --- | --- | --- |
| R 1 | Fabrication and/or falsification | “Fabrication is making up data or results and recording or reporting them; Falsification is manipulating research materials, equipment, or processes, or changing or omitting data or results such that the research is not accurately represented in the research record” [ORI*]. This category includes practice or data described as fraudulent. |
| R 2 | Plagiarism | “Plagiarism is the appropriation of another person’s ideas, processes, results, or words without giving appropriate credit” [ORI*]. Included in this category are concerns about overlap with articles where it is unclear whether plagiarism of others or self-plagiarism is involved. |
| R 3 | Scientific misconduct, type unspecified |  |
| R 4 | Ethical misconduct | Concern about an aspect of a study relating to research ethics, such as gaining ethics approval or participant consent. |
| R 5 | Duplicate publication | Authors published the same article in another journal. |
| R 6 | Overlap | “Multiple publication of the same data or self-plagiarism” [Decullier**]. |
| R 7 | Validity | Concern about the validity of data, methods, or interpretation. |
| R 8 | Peer review or editorial conduct | Concern about editorial or peer reviewer conduct. |
| R 9 | Publisher factor | Article published in error or problem arose because of action by publisher. |
| R 10 | Dispute | A dispute about authorship, permission to publish, or data ownership. |
| R 11 | Reason unspecified |  |
| C 1 | Open | Ongoing editorial deliberation or no conclusion noted. |
| C 2 | Closed | Editorial deliberation concluded (indicated explicitly or inferred by relevant erratum or retraction of publication). |
| C 3 | Ambiguous | Unclear whether editorial deliberation is continuing or concluded. |
| Im 1 | Involves images | The involvement of images is specified. |
| Im 2 | Does not involve images | The involvement of images is not explicitly specified. |
| Adj 1 | Journal adjudication | Journal determined research misconduct (R 1 – 4). |
| Adj 2 | External adjudication | External party determined research misconduct (R 1 – 4). |
| Adj 3 | Journal & external adjudication | Both journal and external party determined research misconduct (R 1 – 4). |
| Adj 4 | Adjudication not stated | Research misconduct found (R 1 – 4), but which party made this determination was not stated. |
| Inv 1 | Official investigation instigated journal concern | Referred to the journal by the employer or funding agency, or the journal became aware of their investigation. |
| Inv 2 | Journal and official investigation, instigation unclear | Both journal and employer/funding agency are investigating, and it is unclear which party instigated the concern. |
| Inv 3 | Journal referred case | Journal referred their concern to the employer, funding agency, or an unspecified type of investigation (such as “independent investigation”). |
| Inv 4 | No official investigation | No official investigation is mentioned. Included here are cases where the journal refers to an investigation by the editors of another journal. |

Footnotes:

* ORI: HHS Office of Research Integrity: <https://ori.hhs.gov/definition-misconduct> [Accessed 3 February 2017]

** Decullier E, Huot L, Samson G, Maisonneuve H. Visibility of retractions: a cross-sectional one-year study. BMC Res Notes. 2013;19:238.
